# LIMK2 Is a Novel Prognostic Biomarker and Correlates With Tumor Immune Cell Infiltration in Lung Squamous Cell Carcinoma

**DOI:** 10.3389/fimmu.2022.788375

**Published:** 2022-02-22

**Authors:** Yongcheng Su, Beibei Xu, Qianwen Shen, Ziyu Lei, Wenqing Zhang, Tianhui Hu

**Affiliations:** ^1^ Cancer Research Center, Xiamen University School of Medicine, Xiamen, China; ^2^ Department of General Surgery, The First Hospital of Xiamen University, School of Medicine, Xiamen University, Xiamen, China; ^3^ Shenzhen Research Institute of Xiamen University, Shenzhen, China

**Keywords:** LIMK2, lung squamous cell carcinoma, prognosis, non-coding RNA, immune infiltrating

## Abstract

Previous research found that LIM domain kinase 2 (LIMK2) expression correlated with a poor prognosis in many cancers. However, its role in lung squamous cell carcinoma (LUSC) has not yet been clarified. Our study aimed to clarify the role of LIMK2 in LUSC prognosis prediction and explore the relationship between LIMK2 and immune infiltration in LUSC. In this study, we first analyzed the expression level and prognostic value of LIMK2 across cancers. Subsequently, we explored the association of LIMK2 expression with immune infiltrating cells and immune checkpoints. our study found that LIMK2 was highly expressed and positively associated with the overall survival of LUSC. Moreover, our study further indicated that LIMK2 expression was significantly negatively correlated with immune cell infiltration and immune checkpoints in LUSC. Finally, we confirmed upstream regulatory noncoding RNAs (ncRNAs) of LIMK2, and the PVT1 and DHRS4-AS1/miR-423-5p/LIMK2 regulatory axes were successfully constructed in LUSC. Put together, LIMK2 is a novel prognostic biomarker and correlates with tumor immune cell infiltration in LUSC, and the expression of LIMK2 is regulated by the PVT1 and DHRS4-AS1/miR-423-5p axes.

## Introduction

Lung cancer (LC) is the second most frequent malignancy after breast cancer, and it is also the principal cause of cancer-related mortality in the world (1). Lung cancer can be divided into small cell lung cancer (SCLC) and non-small cell lung cancer (NSCLC); lung squamous cell carcinoma (LUSC) and lung adenocarcinoma (LUAD) are two subtypes of NSCLC (2). Different subtypes have different clinical characteristics, treatment methods, and prognoses. Although mankind has made significant progress in diagnosis and treatment, the prognosis of lung cancer patients is still not optimistic, which causes at least 1.8 million deaths every year (1). Therefore, finding new therapeutic targets and valuable prognostic markers are urgently needed.

LIM domain kinase (LIMK) is a class of serine/threonine protein kinases (3); previous studies indicated that LIMK1 was highly expressed in numerous malignant tumors and correlated with patients’ poor prognoses (4–8). However, there is little research on the role of LIMK2 in malignant tumors. Our researchers found that LIMK2 was highly expressed in many cancers, such as liver cancer and lung squamous cell carcinoma. The expression level of LIMK2 was positively correlated with the overall survival of some cancers, contrary to previous studies (9–11). Immunotherapy is a novel cancer treatment that has made significant progress in lung cancer, head and neck tumors, lymphoma, and other tumors (12). However, for lung squamous cell carcinoma, immunotherapy cannot achieve the ideal therapeutic effect (13). Our study aimed to further clarify the role of LIMK2 in LUSC prognosis prediction and explore the relationship between LIMK2 and immune infiltration in LUSC.

## Results

### Expression of LIMK2 Across Cancers

**Figure 1:**
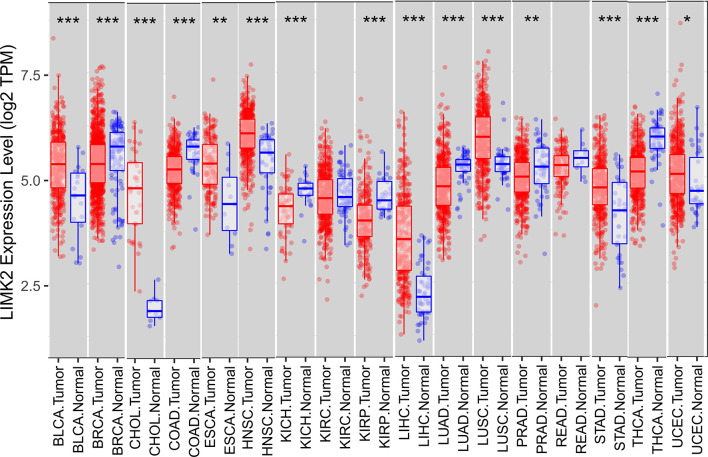
Expression of LIMK2 in 17 human cancer types. *p < 0.05, **p < 0.01, and ***p < 0.001.

**Figure 2:**
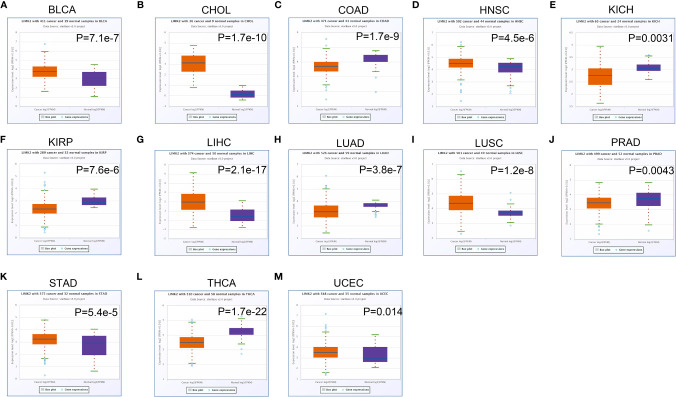
The expression of LIMK2 in BLCA **(A)**, CHOL **(B)**, COAD **(C)**, HNSC **(D)**, KICH **(E)**, KIRP **(F)**, LIHC **(G)**, LUAD **(H)**, LUSC **(I)**, PRAD **(J)**, STAD **(K)**, THCA **(L)**, and UCEC **(M)** based on the StarBASE database.

In this study, we first analyzed the expression level of the LIMK2 gene in human tumors based on the TIMER database. As presented in **Figure 1**, the expression of the LIMK2 gene was significantly upregulated in 7 TCGA tumor types compared with adjacent normal tissues, including BLCA, CHOL, ESEA, HNSC, LIHC, LUSC, STAD, and UCEC, and downregulated in 6 TCGA tumor types, namely, BRCA, COAD, KIRH, KIRP, LUAD, and THCA. Then, we further verified the expression level of LIMK2 in various cancers based on the StarBASE database. As shown in **Figure 2**, in BLCA, CHOL, COAD, LIHC, LUSC, STAD, and UCEC, the expression of LIMK2 was increase remarkably compared with normal tissues. There was a marked decrease in COAD, KICH, KIRP, LUAD, PRAD, and THCA. Based on the StarBASE database and GEPIA database, we can infer that LIMK2 was upregulated in BLCA, CHOL, LIHC, LUSC, STAD, and UCEC and downregulated in COAD, KICH, KIRP, LUAD, and THCA and that LIMK2 may be a key regulatory factor in the above cancers.

### Survival Analysis of LIMK2 Across Cancers

**Figure 3:**
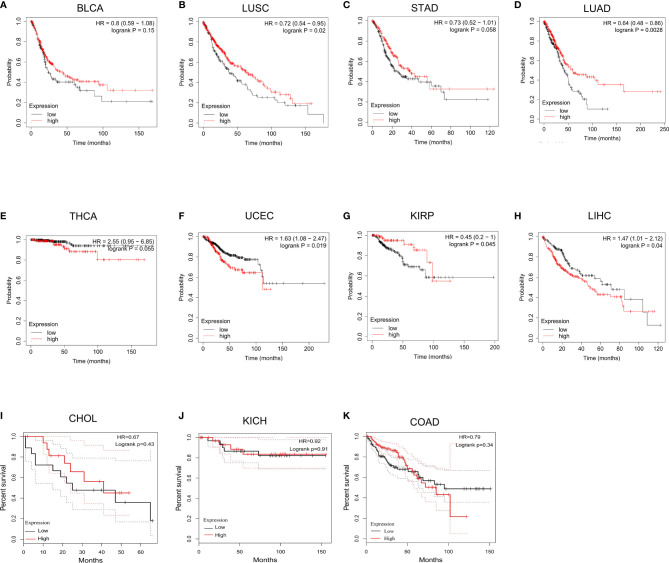
The overall survival analysis for LIMK2 in BLCA **(A)**, LUSC **(B)**, STAD **(C)**, LUAD **(D)**, THCA **(E)**, UCEC **(F)**, KIRP **(G)**, LIHC **(H)**, CHOL **(I)**, and KICH **(J)**, COAD **(K)** based on TCGA database.

To further explore the prognostic value of LIMK2 across cancers, we then performed survival analysis using Kaplan Meier Plotter (http://www.kmplot.com). As shown in **Figure 3**, high expression of LIMK2 was associated with poor prognosis in LIHC and UCEC. However, the high expression of LIMK2 in LUSC, LUAD, and KIRP had a better prognosis. For other cancer types, no significant correlation was observed.

For validation, we downloaded gene expression data and corresponding clinical information for 551 LUSC patients from the TCGA database (http://portal.gdc.cancer.gov). Differential expression analysis revealed that LIMK2 expression level was significantly higher in LUSC, compared with normal tissues (**Supplementary Figure 1A**). Survival analysis was also consistent with all of the previous results, high expression LIMK2 was correlated with better OS and DFS (**Supplementary Figures 1B, C**). And the ROC curves showed that LIMK2 with the power to identify well between controls and LUSC patients (**Supplementary Figure 1D**). In terms of OS and DFS, the area under the curve (AUC) was greater than 0.5, which indicated that LIMK2 has some predictive power in OS and DFS in LUSC patients (**Supplementary Figures 1E, F**).

### Correlation of LIMK2 Expression With Immune Infiltration in LUSC

**Figure 4:**
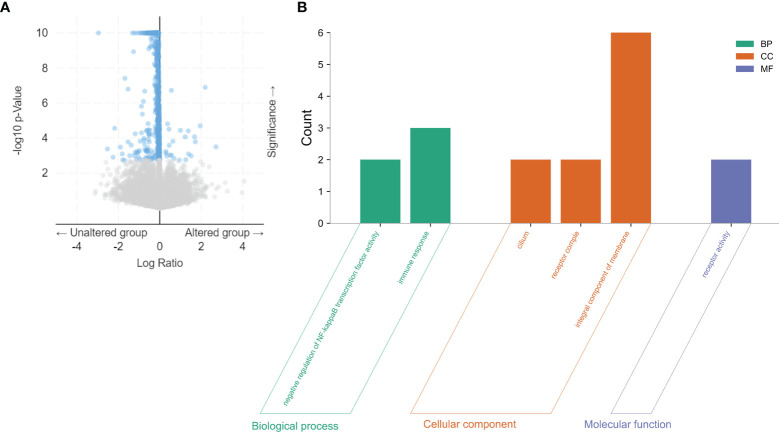
The co-occurrence gene mutations for LIMK2 mutation and wild type in LUSC patients were visualized via volcano plots based on the cBioPortal database **(A)**. GO analysis of coexpressed genes with LIMK2 mutations in LUSC by utilizing the DAVID database **(B)**.

Based on the cBioPortal database, we analyzed the cooccurrence gene mutations between LIMK2 mutation and wild-type patients, which were visualized *via* volcano plots in **Figure 4A**. There was a significant difference in the number of coexisting mutations between LIMK2 mutation and wild type. Then, gene ontology (GO) enrichment analysis of the genes coexpressed with the LIMK2 mutation gene was performed. As presented in **Figure 4B**, biological process (BP) analysis showed that immune response (GO:0006955) and negative regulation of NF-κB transcription factor activity (GO:0032088) were the most important. Similarly, in terms of cell component (CC), there were three remarkably enriched GO terms, including cilium (GO:0005929), integral component of membrane (GO:0016021) and receptor complex (GO:0043235). In conclusion, it is not difficult to speculate that LIMK2 is closely related to the immune response.

**Figure 5:**
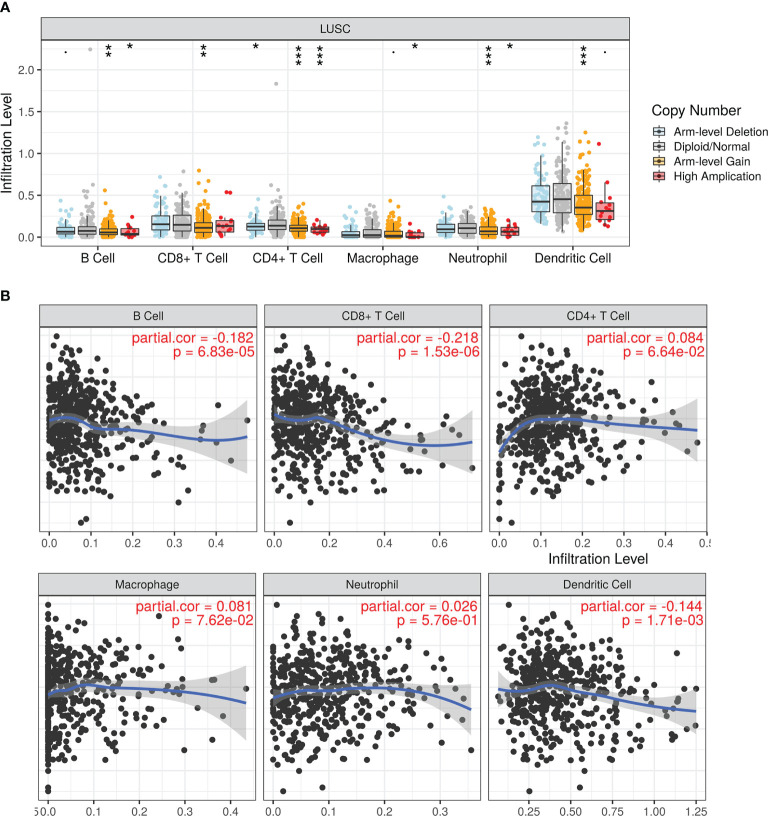
The relationship of immune cell infiltration with LIMK2 levels in LUSC. **(A)** The infiltration level of each immune subset at different copy numbers of LIMK2 in LUSC. **(B)** The correlation of each immune subset infiltration level (B cells, CD8+ T cells, CD4+ T cells, macrophages, neutrophils, and dendritic cells) with the LIMK2 expression level in LUSC.

Next, we analyzed the association between different levels of LIMK2 copy number and the infiltration level of various immune cells in LUSC; as presented in **Figure 5A**, copy number variation was significantly associated with the infiltration level of immune cells in LUSC, showing that LIMK2 could influence LUSC patient outcomes by changing the immune infiltration level. Correlation analysis between LIMK2 expression level and immune cell infiltration level was performed using the TIMER database; the result is depicted in **Figure 5B**. LIMK2 expression was significantly negatively connected with B cells, dendritic cells, and CD8+ T cells in LUSC.

### Correlation Analysis Between LIMK2 Expression and Immune Cell Markers

To further clarify the role of LIMK2 in tumor immunity, correlation analysis between LIMK2 and immune cell markers in LUSC was performed based on the GEPIA database. As inferred from **Table 1**, the LIMK2 expression level was remarkably negatively correlated with immune cell markers, including B cell markers (CD19 and CD79A), CD8+ T cell markers (CD8A and CD8B), M2 macrophage markers (VSIG4 and MS4A4A), and dendritic cell markers (HLA-DPB1, HLA-DRA, and HLA-DPA1), in LUSC. These results further support our inference that LIMK2 expression was significantly negatively correlated with immune cell infiltration in LUSC.

**Table 1:** Correlation analysis between LIMK2 and biomarkers of immune cells in LUSC based on the GEPIA database.

Immune cell	Biomarker	R value	p value
B Cell	CD19	-0.1	0.02*
	CD79A	-0.14	0.0015**
CD8+ T Cell	CD8A	-0.33	5.1e-14***
	CD8B	-0.27	3e-09***
CD4+ T Cell	CD4	-0.038	0.40
M1 macrophage	NOS2	0.13	0.0044**
	IRF5	0.11	0.017*
	PTGS2	0.19	1.6e-05***
M2 macrophage	CD163	-0.067	0.14
	VSIG4	-0.1	0.024*
	MS4A4A	-0.11	0.011*
Neutrophil	CEACAM8	0.13	0.0052**
	ITGAM	0.083	0.07
	CCR7	-0.074	0.10
Dendritic Cell	HLA-DPB1	-0.14	0.0028**
	HLA-DQB1	-0.083	0.07
	HLA-DRA	-0.19	2e-05***
	HLA-DPA1	-0.15	0.00068***
	CD1C	0.087	0.06
	NRP1	0.19	3.1e-05***
	ITGAX	0.093	0.041*

*p value < 0.05; **p value < 0.01; ***p value < 0.001. *Statistically significant differences at p < 0.05, ** statistically significant differences at p < 0.01, *** statistically significant differences at p value < 0.001.

### Association Between LIMK2 and Expression of Immune Checkpoints in LUSC

**Figure 6:**
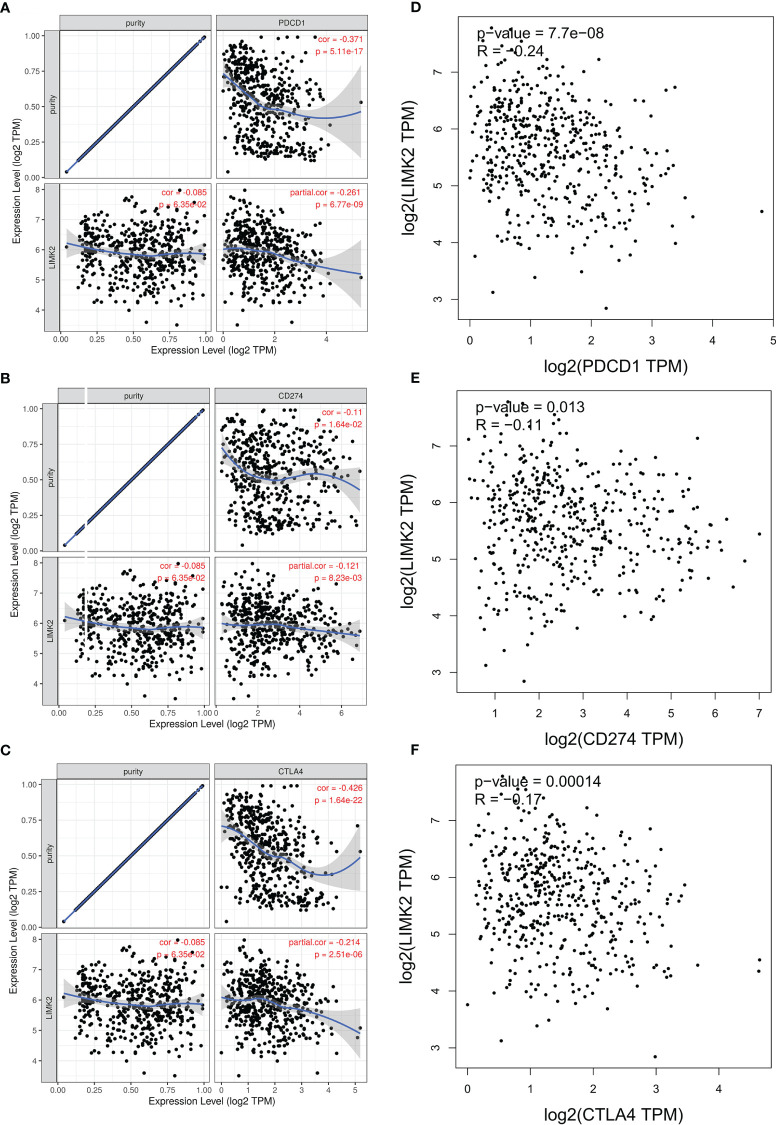
Correlation of LIMK2 expression with PDCD1, CD274, and CTLA-4 expression in LUSC. (A-C) Spearman correlation of LIMK2 with the expression of PDCD1 **(A)**, CD274 **(B)** and CTLA4 **(C)** in LUSC based on the TIMER database. **(D–F)** Correlation of LIMK2 expression with PDCD1 **(D)**, CD274 **(E)**, and CTLA-4 **(F)** expression in LUSC based on the GEPIA database.

Previous studies showed that PDCD1 (PD1), CD274 (PDL1), and CTLA-4 were closely associated with immune escape in the tumor microenvironment. Given the vital role of LIMK2 expression in the immune system, we further examined the relationship between LIMK2 and PDCD1/CD274 and CTLA-4. As shown in **Figures 6A**–**C**, LIMK2 was remarkably negatively correlated with PDCD1, CD274, and CTLA-4 in LUSC; the same results were also observed in the GEPIA database (**Figures 6D**–**F**). The results above demonstrated that LIMK2 could improve the survival of LUSC patients by suppressing the immune response of the tumor microenvironment, which further showed that LIMK2 could be used as a prognostic marker in patients with LUSC.

### Prediction of Upstream Regulatory miRNAs of LIMK2

**Figure 7:**
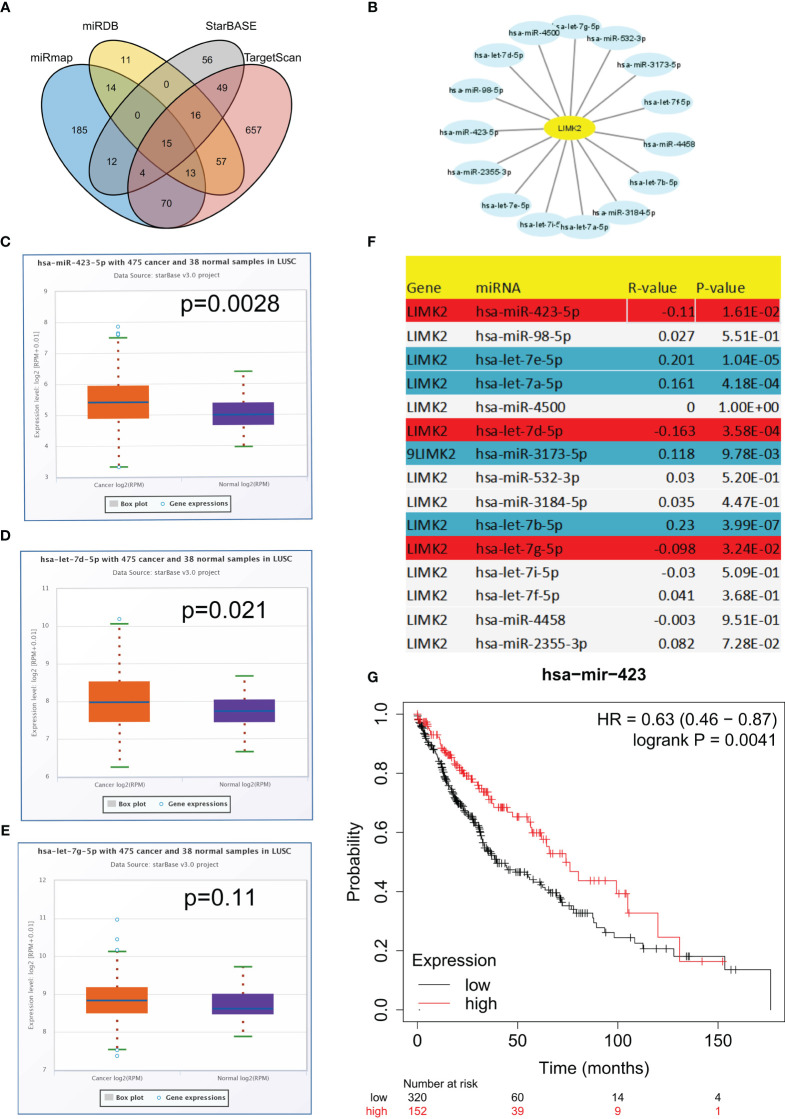
Identification of the potential upstream miRNA targets of LIMK2 in LUSC. **(A)** Venn diagram showing the intersection of upstream regulatory miRNAs of LIMK2. **(B)** The miRNA-LIMK2 regulatory network established by Cytoscape software. **(F)** The correlation between predicted miRNAs and LIMK2 in LUSC analyzed by the STARBASE database. The expression of miR-423-5p **(C)**, let-7d-5p **(D)** and let-7g-5p **(E)** in LUSC compared with control normal samples based on the STARBASE database. **(G)** The prognostic value of miR-423-5p in LUSC based on Kaplan–Meier plotter.

To explore potential upstream regulators of LIMK2, we predicted upstream miRNAs for LIMK2 by using the miMAP, miRDB, StarBASE, and TargetScan databases. As presented in **Figures 7A, B**, a Venn diagram showed the intersection of LIMK2 upstream regulatory miRNAs predicted by the miMAP, miRDB, StarBASE, and TargetScan databases, and 15 miRNAs were considered possible potential regulatory miRNAs (**Figure 7B**). Subsequently, we analyzed the correlation between LIMK2 and 15 miRNAs. The results are shown in **Figure 7F**. Three miRNAs were significantly negatively correlated with LIMK2, including let-7g-5p, let-7d-5p, and miR-423-5p, and four miRNAs, let-7e-5p, let-7a-5p, miR-3173-5p, and let-7b-5p, were remarkably positively correlated with LIMK2. Then, we detected the expression levels of let-7g-5p, let-7d-5p, and miR-423-5p in LUSC; there was a significant difference in the expression of let-7d-5p and miR-423-5p in LUSC (**Figures 7C**–**E**). Finally, we found that miR-423-5p was closely related to the prognosis of LUSC (**Figure 7G**). These findings indicate that miR-423-5p is likely to be the upstream regulatory miRNA of the LIMK2 gene in LUSC.

We first overexpressed miR-423-5p in the lung squamous carcinoma cell line (NCI-H292); subsequently, we explore the effects of miR-423-5p over-expression on the RNA expression level of LIMK2. As showcased in **Supplementary Figure 1A**, the expression of miR-423-5P was significantly increased in the miR-423-5P mimic group; furthermore, prominent downregulation of RNA expression level of LIMK2 was observed compared with the NC-mimic group (**Supplementary Figure 1B**). Conversely, the RNA expression level of LIMK2 was increased with the downregulation of miR-423-5p expression using miR-423-5p inhibitor. Consequently, our experimental results proved that LIMK2 was a reliable target of miR-423-5p (**Supplementary Figures 2A, B**).

### Prediction of Upstream lncRNAs of miR-423-5p

**Figure 8:**
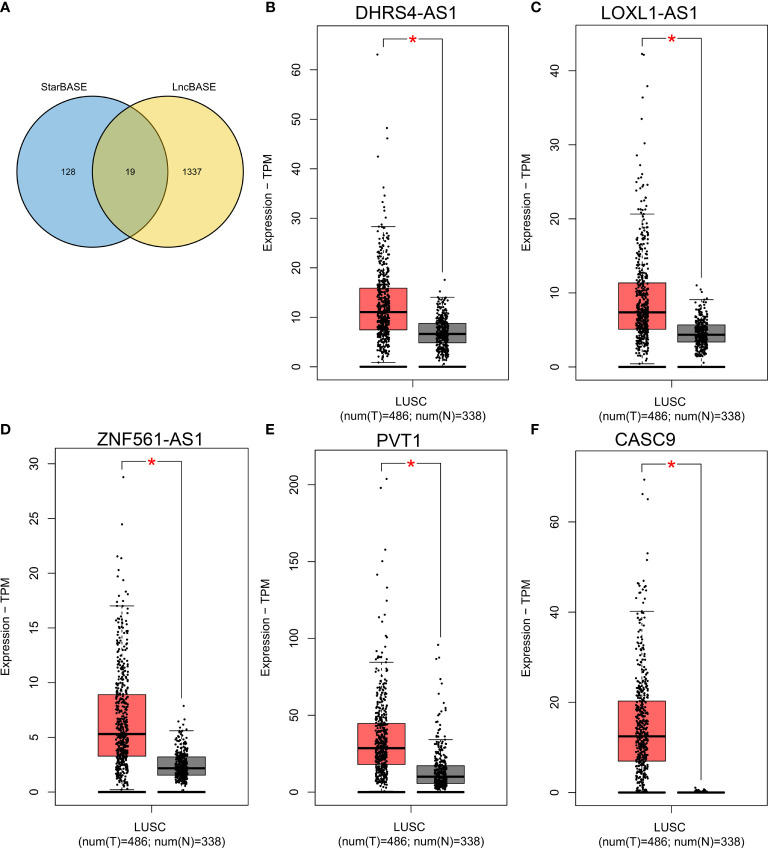
Expression analysis of upstream lncRNA targets of miR-423-5p in LUSC. **(A)** Venn diagram shows the overlap of potential lncRNA targets from the STARBASE and lncBASE databases. **(B–F)** The expression of DHRS4-AS1 **(B)**, LOXL1-AS1 **(C)**, ZNF561-AS1 **(D)**, PVT1 **(E)** and CASC9 **(F)** in TCGA LUSC compared with TCGA normal and GTEx data. *p < 0.05.

**Figure 9:**
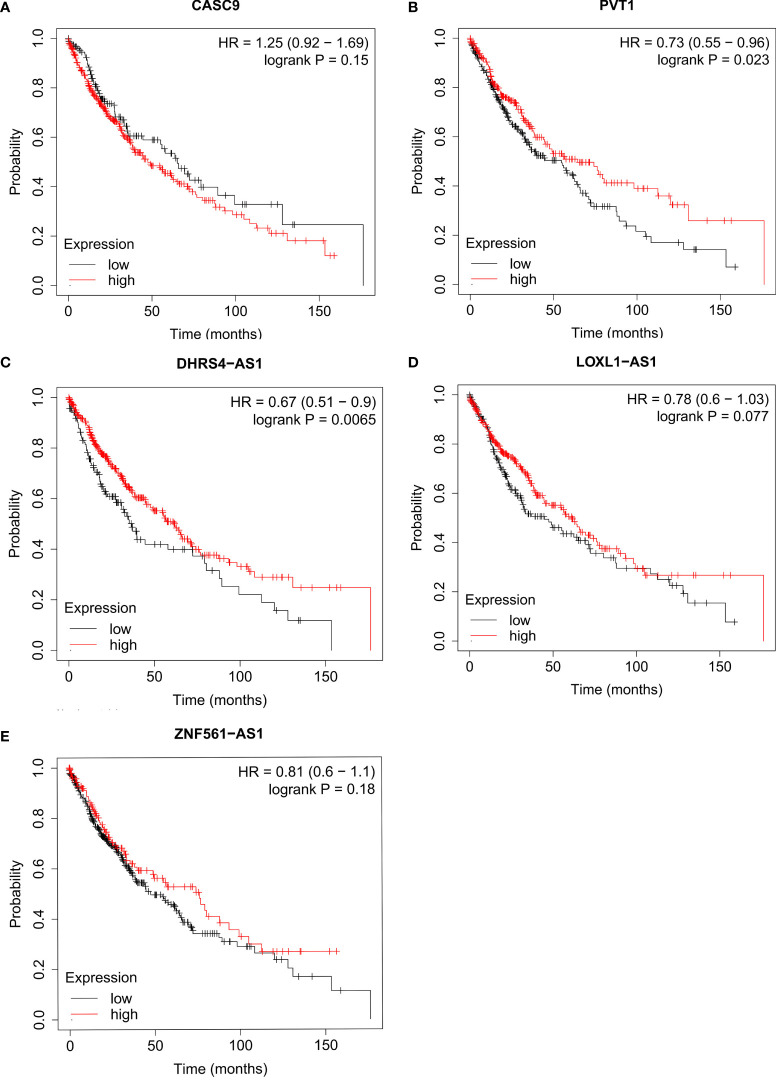
Survival analysis for upstream lncRNA targets of miR-423-5p in LUSC. The overall survival for CASC9 **(A)**, PVT1 **(B)**, DHRS4-AS1 **(C)**, LOXL1-AS1 **(D)** and ZNF561-AS1 **(E)** in TCGA LUSC compared with TCGA normal and GTEx data.

Then, we further predicted the potential regulatory lncRNA of miR-423-5p with the help of StarBASE and LncBASE. As shown in **Figure 8A**, only the lncRNAs appearing in both databases were considered potential regulatory molecules of miR-423-5p. Subsequently, we also determined the expression levels of these lncRNAs in LUSC based on the GEPIA database. Only five lncRNAs showed significant differences in LUSC, including DHRS4-AS1, LOXL1-AS1, ZNF561-AS1, PVT1, and CASC9 (**Figures 8B**–**F**). Survival analysis for the above five lncRNAs was performed using Kaplan–Meier Plotter; the results are presented in **Figure 9**. DHRS4-AS1 and PVT1 were closely related to the survival of LUSC patients. Based on the above results, it is reasonable to speculate that PVT1 and DHRS4-AS1 are potential upstream regulatory lncRNAs of the miR-423-5p/LIMK2 axis in LUSC.

## Discussion

NSCLC is one of the leading causes of cancer death (14), although great progress has been made in treatment, especially in targeted therapy for LUAD, which has greatly improved the prognosis of advanced LUAD. However, for LUSC, there is currently no better treatment; targeted therapy seems useless for LUSC (15). Searching for prognostic markers and novel therapeutic targets for LUSC appears crucial.

Based on the TIMER and GEPIA databases, this study found that LIMK2 was highly expressed in LUSC. Meanwhile, the survival analysis also showed that high LIMK2 expression was associated with a better prognosis in LUSC, suggesting that LIMK2 may become a prognostic marker for LUSC. However, previous studies have demonstrated that LIMK2 is correlated with poor prognosis in many cancers, including bladder cancer (16), breast cancer (17), and prostate cancer (18). Both LIMK1 and LIMK2 belong to the LIMK family; despite their structural similarities, the LIMK1/LIMK2 may have different roles in cancer development and progression. A study noted by Zhang et al. indicated that LIMK2 could inhibit tumor cell proliferation and migration; reduced LIMK2 expression would activate the Wnt- signaling pathway, promoting tumor progression (19). Lourenço et al. also showed that patients with high LIMK2 expression had a significantly better prognosis than low LIMK2 expressing patients (20). Furthermore, LIMK 2b, as an alternative transcript of LIMK, could function as a tumor suppressor *via* G2/M arrest mediated by th p53 signaling pathway (21). The above results are consistent with the conclusion of our study, which provides further evidence for the anti-tumor effect of LIMK2, although the exact mechanism is poorly understood.

To explore the role of LIMK2 in patients with LUSC, we further analyzed the comutated genes between LIMK2 mutation and wild type in LUSC patients. Then, we performed GO analysis for the genes comutated with LIMK2. The GO analysis results revealed that genes comutated with LIMK2 were involved in the immune response; therefore, it is reasonable to suspect that LIMK2 may be associated with the tumor immune response. Tumor immune cell infiltration is well known to influence the treatment outcomes and prognosis of patients (22–24); studies have also shown that inhibition of immune checkpoints can reverse tumor immune evasion and then improve patient outcomes (25, 26). With the aid of the TIMER database, we found that LIMK2 expression levels were significantly negatively correlated with B cells, CD8+ T cells, and dendritic cells in LUSC.

DCs are a class of highly efficient antigen-presenting cells, which have consistently been recognized to be related to CD8+ T cell activation and antitumor immunity (27, 28). Whereas different subsets and different functional states will lead to changes in the relationship between DCs and tumor prognosis (29), for instance, plasmacytoid DCs (pDCs) are linked to poor prognosis in breast cancer and ovarian cancer patients (30, 31). On the other hand, pDCs have been shown to play antitumor effects by inducing apoptosis of tumor cells (30). Similarly, except for renal cell cancer (RCC) (32) and prostate cancer (33), CD8 T cell was regarded as a favorable prognostic marker and had been validated a variety of tumors, including ovarian cancer (34), liver cancer (35), breast cancer (36), and lung squamous cell carcinoma (37, 38), in the study of Romain Remark et al., the expression level of VEGF was positively associated with CD8 T immune cell infiltration in RCC, which might explain why high CD8 T cell infiltration leads to poor prognosis (32). Likewise, Leclerc et al. also showed that CD73 expression would function to suppress immune surveillance mediated by CD8+cells and then turn them into cancer-promoting factor (39). The function of B cells in tumors remains controversial (40), Pauline Andreu et al. indicated that B cells could promote cancer development by activating Fcγreceptors (41). In our study, high expression LIMK2 was negatively correlated with DCs, B cells, and CD8 T cells infiltration in LUSC patients. Furthermore, LIMK2 was also remarkably negatively associated with PDCD1, CT274, and CTLA-4 in LUSC. All the above results may explain why immunotherapy could not act efficiently in LUSC.

The competitive endogenous RNA (ceRNA) network, a gene expression regulation hypothesis proposed by Salmena (42), is involved in the development of several tumors. Thus far, the ceRNA network has been widely recognized. In this study, we tried to determine the upstream miRNAs of LIMK2 based on the ceRNA hypothesis; with the help of the miMAP, miRDB, StarBASE, and TargetScan databases, we found that miR-423-5p is likely to be the upstream regulatory miRNA of the LIMK2 gene in LUSC. Studies have demonstrated that miR-423-5p is associated with tumor development and poor prognosis of patients (43–47). Then, upstream regulatory lncRNAs of miR-423-5p/LIMK2 were predicted by the StarBASE and lncBASE databases. Finally, PVT1 and DHRS4-AS1 were considered the most likely potential upstream regulatory lncRNAs of the miR-423-5p/LIMK2 axis in LUSC. In summary, PVT1 and DHRS4-AS1/miR-423-5p/LIMK2 ceRNA networks were successfully constructed in LUSC.

Put together, LIMK2 could be used as a prognostic biomarker and was associated with a better prognosis in LUSC. Our study indicated that LIMK2 might function as a tumor suppressor by decreasing tumor immune infiltration and restricting the expression of immune checkpoints. Moreover, we further identified PVT1 and DHRS4-AS1/miR-423-5p as potential upstream regulators of LIMK2. Naturally, our findings have significant clinical implications and need to be validated in clinical and basic studies.

## Materials and Methods

### Datasets

The gene expression file (FPKM value) and corresponding clinical data of 551 lung squamous cell carcinomas patients were retrieved from the TCGA-LUSC database (http://portal.gdc.cancer.gov).

### Cell Lines and Cell Culture

Lung squamous cell carcinoma cell lines NCI-H292 were obtained from the Chinese Academy of Sciences (Shanghai, China) Cell Bank of Type Culture Collection. NCI-H292 cells were grown in 1640 with 10% fetal bovine serum (FBS) and cultured at 37°C in a 5% CO2 incubator.

### Transfection

NCI-H292 cells were inoculated into 6-well culture plates. After 24 h, prepare complexes as follows for each transfection sample: mimic NC, inhibitor NC, miR-423-5p mimic, and miR-423-5p as required were transfected with the liposome transfection reagent Lipofectamine 2000 according to the manufacturer’s recommendations. After 6 h, the medium was replaced as 1640 with 10% fetal bovine serum (FBS). Cells were cultured for 18 h to test for transgene expression. The mimics, inhibitor, and primer sequences are listed in **Supplementary Tables 1** and **2**, respectively.

### RT-Quantitative PCR (qPCR)

According to the manufacturers’ instructions, total RNA was extracted with the Trizol (Invitrogen) reagent. 1 μg of mRNA was reversely transcribed to cDNA by the reverse transcription system. Then, the mRNA expression levels of target genes were detected using the LightCycler 96 real-time PCR system (Roche Diagnostics GmbH, Mannheim, Germany) with the SYBR Premix EX Taq (YEASEN Biotech, Shanghai, China). The GAPDH gene level was selected as an internal control.

### TIMER Database Analysis

TIMER is a database that is capable of analyzing the correlation between tumors and the immune system (48)(https://cistrome.shinyapps.io/timer). The expression analysis of LIMK2 across cancers was conducted by using the TIMER database. The correlation between LIMK2 expression level and immune cell infiltration level and the immune checkpoint expression level in LUSC were also analyzed based on the TIMER database. P values of <0.05 (P < 0.05) were statistically significant.

### StarBASE Database Analysis

The StarBASE database (http://starbase.sysu.edu.cn) is an online platform that provides miRNA-mRNA and miRNA-lncRNA interactions verified in the laboratory (49). Our study was used to predict the potential upstream miRNA or lncRNA targets of LIMK2 and determine the expression level of LIMK2 and upstream miRNA or lncRNA.

### Kaplan–Meier Plotter Analysis

The Kaplan–Meier plotter (http://kmplot.com/analysis) is an online database. Its primary purpose is a meta-analysis-based discovery and validation of survival biomarkers (50). Survival analysis of the LIMK2 gene and its upstream lncRNA or miRNA targets in LUSC was conducted by utilizing Kaplan–Meier plotter.

### cBioPortal Database Analysis

Based on the cBioPortal data platform (51)(http://cbioportal.org), we analyzed the cooccurrence gene mutations between LIMK2 mutation and wild-type patients.

### DAVID Database Analysis

The DAVID database (52) (https://david.abcc.ncifcrf.gov) was utilized to perform gene ontology (GO) analysis on the genes comutated with LIMK2 in LUSC.

### GEPIA Database Analysis

The GEPIA database is a web-based tool that can perform gene differential expression analysis and correlation analysis (53), based on the GEPIA database. We explored the correlation between LIMK2 and biomarkers of immune cells and immune checkpoints in LUSC.

### Prediction of Potential Upstream miRNA/lncRNA of LIMK2

Open-source databases consisting of miRmap (54), miRDB (55), StarBASE (49) and TargetScan (56) databases were utilized to identify the potential upstream miRNA/lncRNA targets of LIMK2 in LUSC. Only the miRNAs/lncRNAs that appeared simultaneously in the above four databases were considered eligible upstream miRNAs/lncRNAs of LIMK2.

### Statistical Analysis

Statistical analysis was performed using the online database mentioned above. Correlation coefficients were obtained using the Spearman correlation method. Different significance levels were used: *p value < 0.05; **p value < 0.01; and ***p value < 0.001 were considered statistically significant. For Kaplan–Meier plots, a log-rank p value <0.05 was considered significant. Data visualization was performed by R software.

## Data Availability Statement

The original contributions presented in the study are included in the article/**Supplementary Material**. Further inquiries can be directed to the corresponding author.

## Author Contributions

Conceptualization, YS and TH. Data curation, analysis and validation, YS, BX, QS, WZ, and ZL. Writing—original draft, YS and TH. Writing—review and editing, BX and TH. All authors contributed to the article and approved the submitted version.

## Funding

This work was supported by the grants from Shenzhen Science and Technology Program (JCYJ20210324121802008), the Natural Foundation of Fujian Province (2021R1001003, 2021J011343), China Postdoctoral Science Foundation (2021M691895).

## Conflict of Interest

The authors declare that the research was conducted in the absence of any commercial or financial relationships that could be construed as a potential conflict of interest.

## Publisher’s Note

All claims expressed in this article are solely those of the authors and do not necessarily represent those of their affiliated organizations, or those of the publisher, the editors and the reviewers. Any product that may be evaluated in this article, or claim that may be made by its manufacturer, is not guaranteed or endorsed by the publisher.
