# Supplementary material for: LIMK2 Is a Novel Prognostic Biomarker and Correlates With Tumor Immune Cell Infiltration in Lung Squamous Cell Carcinoma
Source: Front Immunol. 2022 Feb 22;13:788375. doi: 10.3389/fimmu.2022.788375 (PMC8902256; doi:10.3389/fimmu.2022.788375)
Supplement: Supplementary file 1 [file DataSheet_1.docx]

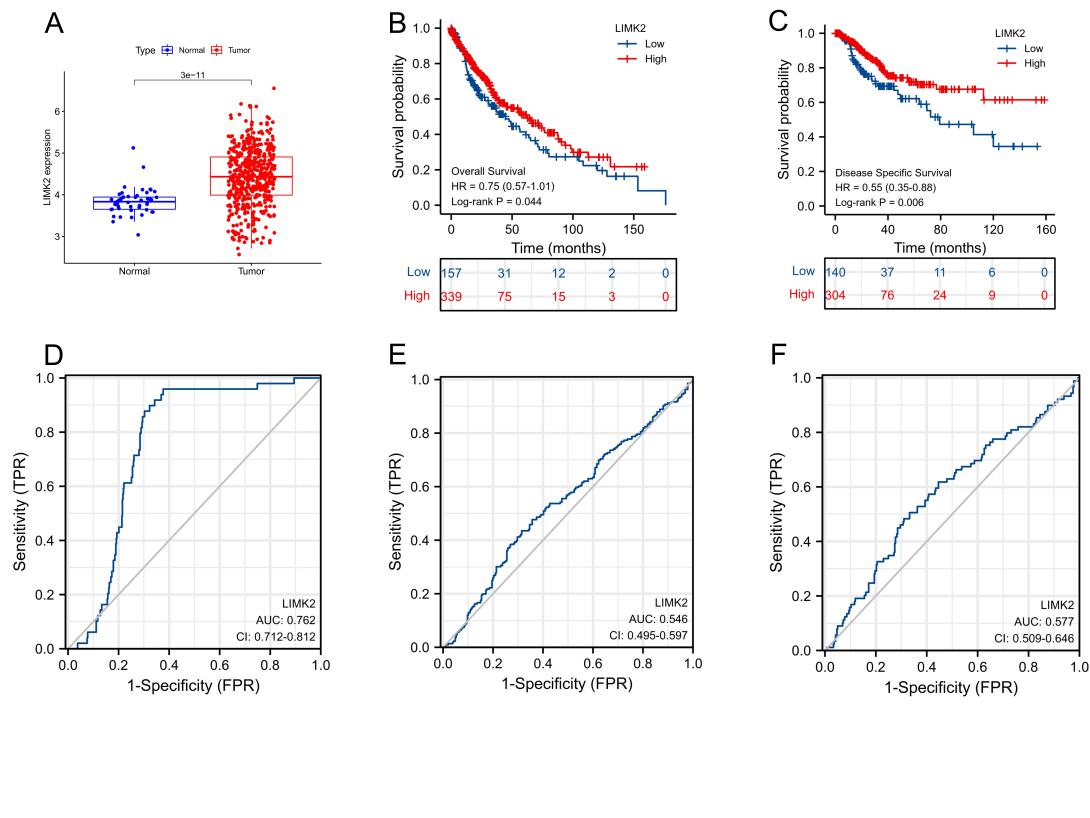


**Supplementary figure 1.** The expression of LIMK2 in LUSC based on the TCGA-LUSC dataset (A). The OS (B) and DFS (C) for LIMK2 in LUSC patients. ROC curve analysis for LUSC prediction (D), OS prediction (E), and DFS prediction (F).

*
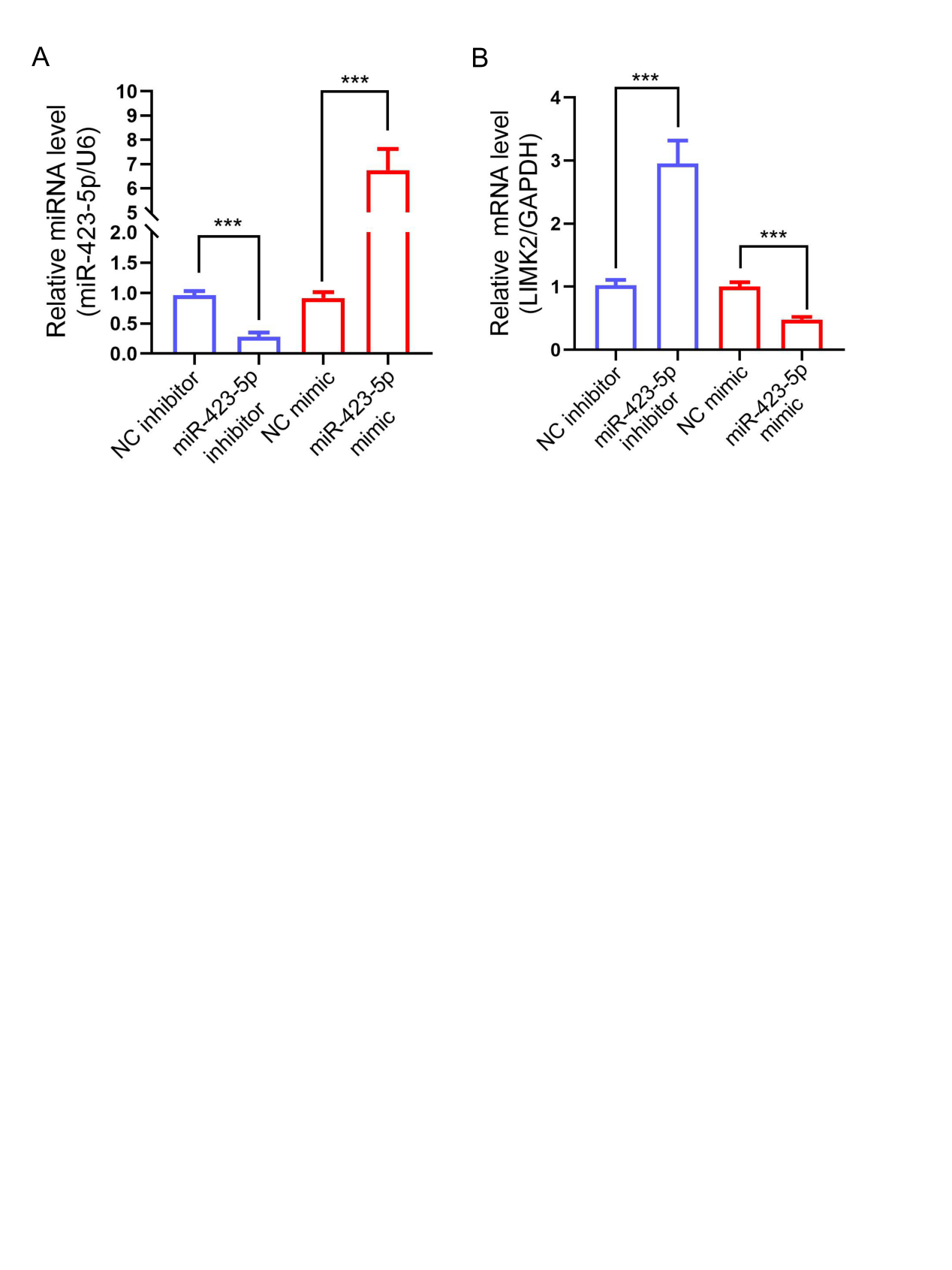
*

**Supplementary figure 2.** RT-PCR analysis of the relative expression of miR-423-5p in lung squamous carcinoma cells (NCI-H292) transfected with miR-423-5p inhibitor or NC-inhibitor, and miR-423-5p mimic or NC-mimic (A). Determination of the LIMK2 expression level in miR-423-5p mimic and miR-423-5p inhibitor treated NCI-H292 cells (B).

**Supplementary Table 1. Mimics/inhibitor sequences list**

|  | **Sequence** |
| --- | --- |
| mimic NC | UUCUCCGAACGUGUCACGUTT |
| inhibitor NC | CAGUACUUUUGUGUAGUACAA |
| miR-423-5p mimic | UGAGGGGCAGAGAGCGAGACUUU |
| miR-423-5p inhibitor | AAAGUCUCGCUCUCUGCCCCUCA |

**Supplementary Table 2. Primer sequences list**

|  | **Sequence** |
| --- | --- |
| miR-423-5p | Forward: ACGTGAGGGGCAGAGAGC  Reverse: GTGCAGGGTCCGAGGT |
| U6 | Forward: CAGCACATATACTAAAATTGGAACG  Reverse: ACGAATTTGCGTGTCATCC |
| LIMK2 | Forward: GGATTCCCTCACCAACTGGTA  Reverse: AGCCACCATAAAAGGCCCTG |
| GAPDH | Forward: TGCACCACCAACTGCTTAGC  Reverse: GGCATGGACTGTGGTCATGAG |

**Supplementary Table 3. Abbreviations are as above**

| BLCA | Bladder Urothelial Carcinoma |
| --- | --- |
| BRCA | Breast invasive carcinoma |
| CHOL | Cholangiocarcinoma |
| COAD | Colon adenocarcinoma |
| ESCA | Esophageal carcinoma |
| HNSC | Head and Neck squamous cell carcinoma |
| KICH | Kidney Chromophobe |
| KIRC | Kidney renal clear cell carcinoma |
| KIRP | Kidney renal papillary cell carcinoma |
| LIHC | Liver hepatocellular carcinoma |
| LUAD | Lung adenocarcinoma |
| LUSC | Lung squamous cell carcinoma |
| PRAD | Prostate adenocarcinoma |
| READ | Rectum adenocarcinoma |
| STAD | Stomach adenocarcinoma |
| THCA | Thyroid carcinoma |
| UCEC | Uterine Corpus Endometrial Carcinoma |
| HNSC | Head and Neck squamous cell carcinoma |
| TCGA | The Cancer Genome Atlas |
| GSEA | Gene set enrichment analysis |
| LASSO | Least absolute shrinkage and selection operator |
| ROC | Receiver operating characteristic |
| CTLA-4 | Cytotoxic lymphocyte associated antigen-4 |
| PD-1 | Programmed cell death protein 1 |
| PD-L1 | Programmed death-ligand 1 |
